# Method for Elucidating the Structural Evolution of a Nanoscale Release Layer in Double Copper Foils Under Thermal Exposure

**DOI:** 10.3390/ma18143316

**Published:** 2025-07-14

**Authors:** Rutuja Bhusari, Julien Bardon, Jérôme Guillot, Adrian-Marie Philippe, Sascha Scholzen, Zainhia Kaidi, Frédéric Addiego

**Affiliations:** 1 Structural Composites Unit, Luxembourg Institute of Science and Technology, 5 Avenue des Hauts-Fourneaux, L-4362 Esch-sur-Alzette, Luxembourg; rutujabhusari05@gmail.com (R.B.); julien.bardon@list.lu (J.B.); 2 Advanced Analyses and Support Unit, Luxembourg Institute of Science and Technology, 5 Avenue des Hauts-Fourneaux, L-4362 Esch-sur-Alzette, Luxembourg; jerome.guillot@list.lu (J.G.); adrianmarie.philippe@list.lu (A.-M.P.); 3 Circuit Foil Luxembourg, 6 Salzbaach, L-9559 Wiltz, Luxembourg; sascha.scholzen@circuitfoil.com (S.S.); zainhia.kaidi@circuitfoil.com (Z.K.)

**Keywords:** copper foil, release layer, nanomaterial, multiscale characterization, thermal exposure

## Abstract

Double ultrathin copper foils (DTH), widely used for producing conductive tracks in electronics, consist of an ultrathin copper functional foil (FF), a nanometric release layer (RL), and an ultrathin copper carrier foil (CF). Achieving stable release strength of the CF during DTH lamination remains a key challenge, largely due to limited knowledge about the structure of the RL. In this study, a comprehensive characterization methodology is proposed to investigate the physico-chemical structure of a chromium-based RL, both before and after thermal exposure at 230 °C. Peel-off testing, X-ray photoelectron spectroscopy (XPS), atomic force microscopy (AFM), and transmission electron microscopy (TEM) were employed. The main structural transformation identified is the oxidation of the RL at the FF–RL interface, resulting in the formation of a chromium oxide layer. This transformation may underlie the significant increase in release strength, which rises from 5.9 N/m before thermal exposure to 163 N/m afterward.

## 1. Introduction

Miniaturization is receiving increasing attention in the electronics industry due to its potential to reduce material usage, integrate multiple functionalities, contribute to weight reduction in transportation, and simplify users’ daily lives. In this context, ultrathin copper foils produced by electroplating are widely used to create the conductive tracks in printed circuit boards (PCBs) for smartphones, tablets, and laptops. However, these foils—typically less than 12 µm thick—are not self-supporting [1]. To overcome this limitation, modern technologies employ a double-layered ultrathin copper foil (DTH), which consists of a functional foil (FF)—the ultrathin copper layer—and a thicker carrier foil (CF), typically several tens of micrometers thick [2]. These two layers are separated by a nanometer-scale release layer (RL), generally thinner than 30 nm. One step for the manufacturing of the conductive track involves laminating this double foil onto the PCB substrate. During this process, the DTH foil undergoes lamination onto the PCB under pressure and temperatures up to 350 °C [1]. This causes the FF to adhere permanently to the insulating PCB substrate, while the CF is peeled off [1,3]. The RL enables this separation by providing low adhesion to copper, being peelable, and maintaining stability during storage and lamination [4]. Additionally, the RL can serve as a diffusion barrier to prevent copper migration between the FF and CF layers [4]. Various RL designs have been developed, ranging from single-layer to multi-layer configurations. For instance, a peelable metal layer may be sandwiched between two other metals to facilitate plating [5,6]. Release layers can be composed of organic materials [4,5], metal oxides [1,7], metal alloys [8,9], metal phosphates [10], amorphous metals [11], or combinations of these. In the case of amorphous metal RLs, electroplating can be carried out in the presence of carbon or oxygen to disrupt the metal atom arrangement, resulting in an amorphous structure. This structure weakens van der Waals interactions with the copper foils, improving peelability [11].

One major challenge in the development of DTH products is the characterization of the initial structure of the release layer (RL) and its evolution under heat and pressure conditions that simulate the lamination process. Due to the nanometer-scale thickness of RLs, only advanced nanoscale characterization techniques are suitable for this purpose [12]. To the best of our knowledge, no comprehensive structural investigations of RLs in DTH systems have been reported in the literature, which constitutes the primary focus of the present work. Currently, the validation of these products relies solely on macroscopic measurements, specifically the release strength of the carrier foil (CF) determined via peel-off tests as a function of DTH design and thermal exposure. For instance, as a typical validation criterion, the release strength must generally exceed 20 N/m to ensure adequate bonding while remaining below 100 N/m to facilitate efficient peeling [1].

In this paper, we propose a method for resolving the structure of the release layer (RL) using a multiscale approach based on lateral resolution. The centimeter scale is first addressed by measuring the release strength of the DTH product through peel-off testing. Next, the micrometer scale is examined using X-ray photoelectron spectroscopy (XPS) on the debonded surfaces of the DTH structure. Finally, the nanometer scale is investigated using two complementary techniques for cross-sectional analysis: atomic force microscopy (AFM) operated in nanomechanical mode and transmission electron microscopy (TEM) with scanning transmission electron microscopy–energy dispersive X-ray spectroscopy (STEM-EDS). Together, these techniques provide a comprehensive characterization: peel-off testing yields the macroscopic release strength; XPS reveals the elemental composition and chemical states at the surface; AFM provides cross-sectional morphological imaging and elastic modulus mapping; TEM offers high-resolution electron imaging; and STEM-EDS enables elemental analysis of the cross-section. For this study, a DTH product incorporating an amorphous chromium-based RL—referred to as DTH-Cr and manufactured by Circuit Foil Luxembourg—was selected for analysis in both its as-fabricated state and after thermal exposure. This product was chosen due to its relatively simple RL structure and composition, which facilitates the development and validation of the characterization methodology. It is important to note that chromium-based RLs are no longer in use due to the toxicity of hexavalent chromium present in the electroplating electrolyte. However, the characterization methodology developed in this work is designed to be applicable to modern DTH products with more complex RL designs.

## 2. Experimental Section

### 2.1. Material

**Figure 1:**
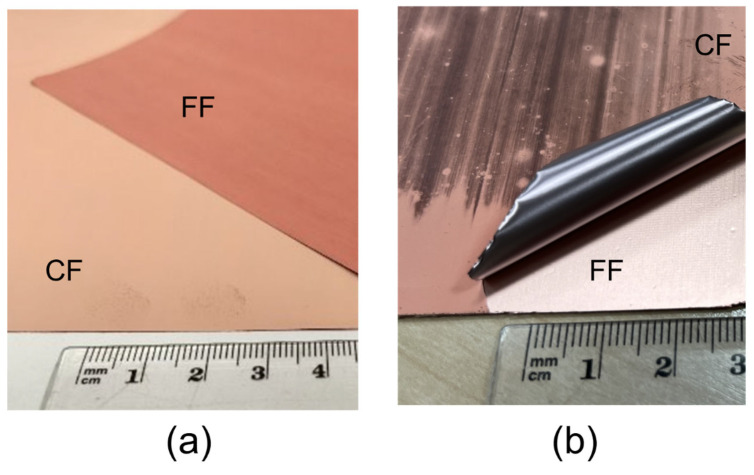
(**a**) Photograph of a DTH foil showing the two copper foils: the functional foil (FF) with a matte surface and the carrier foil (CF) with a shiny surface, attributed to differences in surface roughness. (**b**) Photograph of the DTH-Cr sample laminated onto a prepreg substrate after peeling off the CF.

The DTH-Cr sample investigated in this study (Figure 1a,b) was manufactured by Circuit Foil Luxembourg (Wiltz, Luxembourg) using an industrial electroplating production process. This process consists of three main steps: (1) electroplating the carrier foil (CF) to a thickness of 20 µm, (2) electroplating the release layer (RL) onto the CF, and (3) electroplating the functional foil (FF), with a thickness of 2 µm, onto the RL. The metal electroplating process involves applying a potential difference to an electrolyte solution containing metal ions, causing their reduction and deposition as a solid metal layer. This is achieved using specialized electroplating drums that include both anode and cathode components to facilitate the electrochemical reaction. For structural analysis, it is important to note that the copper in the CF exhibits larger grain sizes compared to that in the FF. Scanning electron microscope (SEM) analysis of DTH-Cr cross-sections showed that the grain size was 1.62 ± 0.74 µm for the CF and 0.80 ± 0.40 µm for the FF. Grain size was determined using ImageJ software (version 1.54g; developed by Wayne Rasband and contributors, National Institutes of Health, Bethesda, MD, USA) and represents the average between the maximum length and width of each grain.

Thermal exposure was conducted by placing the DTH-Cr sample between two metallic plates, which were heated to 230 °C for 110 min in air using a manual hot press (Carver 3851, Carver Press, Wabash, IN, USA). Minimal pressure was applied during this process to avoid influencing the structural evolution of the materials. After exposure, the DTH-Cr foils were removed from the plates and allowed to cool to room temperature in air (21 ± 2 °C). Additional thermal exposure experiments were also performed at 170 °C and 200 °C (each for 110 min), and the corresponding data are provided in the Supplementary Section.

### 2.2. Characterization

The release strength of the DTH-Cr was measured using a 90° peel test system (Instron, Norwood, MA, USA) conducted at room temperature (21 ± 2 °C) on samples with a width of 1 inch. Peeling was performed over a length of 0.11 m, and the average release strength was calculated and reported in units of N/m.

X-ray photoelectron spectroscopy (XPS) measurements were performed using an Axis Ultra DLD system (Kratos Analytical Ltd., Stretford, UK) on both counter surfaces obtained after manual peeling of the DTH-Cr sample (Figure 1b). The peeled surfaces are hereafter referred to as DTH-Cr-FF for the functional foil side and DTH-Cr-CF for the carrier foil side. The spectrometer was equipped with a monochromatic Al Kα X-ray source (energy = 1486.6 eV) operated at 150 W, providing an effective analysis depth of up to approximately 10 nm. Spectra were acquired over an area of 700 µm × 300 µm using a pass energy of 20 eV, which corresponds to a full width at half maximum (FWHM) of 0.6 eV for the Ag 3d_5_/_2_ peak on a sputter-cleaned silver standard. Elemental quantification and peak fitting were carried out using CasaXPS software (version 2.3.22).

**Figure 2:**
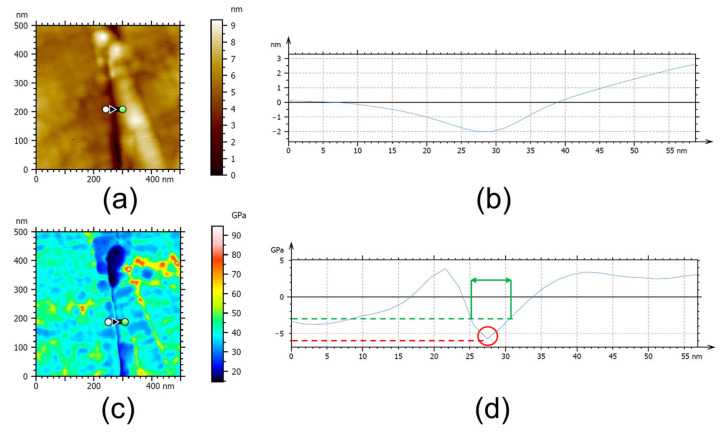
Example of AFM analysis of the RL in the case of untreated DTH-Cr, including (**a**) a topography image, (**b**) an extracted topography profile from (**a**), (**c**) an elastic modulus image, and (**d**) an extracted elastic modulus profile from (**c**). Please note that the profile shown in (**d**) is presented in relative units, with zero corresponding to the average elastic modulus value in image (**c**), which is taken as the elastic modulus of copper. Red and green lines indicate the regions used for evaluating the RL elastic modulus (red circle) and RL thickness (green double arrow), respectively.

For AFM observation, the sample cross-section was finely polished with a cryo-ultramicrotome EM FC6 from Leica (Wetzlar, Germany). More precisely, small samples with the typical dimensions of 5 mm × 5 mm were cut from the DTH-Cr foil and held vertically in a special sample holder, enabling it to be used for both the cryo-ultramicrotome and AFM stages without removing the sample. Thin slices of foil materials were removed by using a diamond knife (reference Cryo 35° from Diatome (Port, Switzerland)) at room temperature (21 ± 2 °C) to achieve a relatively flat surface on the sample surface. It is important to note that this procedure must be performed carefully to avoid debonding the DTH-Cr during the slicing operation. Optical microscope observations were performed to verify that no debonding happened prior to AFM analysis. AFM imaging was performed using an MFP-3D Infinity instrument (Asylum Research, Santa Barbara, CA, USA) operated in amplitude-modulated–frequency-modulated (AM-FM) mode [13] to simultaneously acquire topography and elastic modulus contrast images of the DTH-Cr cross-section. Topography and elastic modulus profiles can then be extracted from the images (see Figure 2a,b for the topography and Figure 2c,d for the elastic modulus). Silicon cantilevers (model AC160TS-R3, Olympus (Tokyo, Japan)) with a nominal spring constant of 26 N/m and a tip radius of 7 nm were used. All measurements were conducted under ambient conditions (21 ± 2 °C, relative humidity ~50%) using a standard air cantilever holder. Images were acquired over areas ranging from 0.8 µm × 0.8 µm to 1.5 µm × 1.5 µm at a resolution of 256 pixels × 256 pixels, with a scan rate of 2 Hz. The actual spring constants of the cantilevers (20–30 N/m) were calibrated using the GetReal Automated Probe Calibration feature (Asylum Research, Santa Barbara, CA, USA), which also determined the deflection sensitivity. The first and second resonance frequencies of the cantilevers were approximately 320 kHz and 1.8 MHz, respectively. To maintain repulsive intermittent contact, the free oscillation amplitude (A_0_) was selected to produce a phase shift close to 60°, and the setpoint amplitude was adjusted to A_set point_/A_0_ = ca. 0.75. Under these conditions, the maximum indentation depth was estimated to be approximately 1 nm [13]. For both the reference and thermally treated DTH-Cr samples, two key structural parameters were extracted: (1) the elastic modulus of the release layer (RL), defined as the minimum modulus value within the RL region, and (2) the RL thickness, defined as the distance between the midpoints of the modulus transitions from copper (CF and FF) to the RL. The method is illustrated in Figure 2d. These values were averaged over a minimum of 5–10 profiles per sample, based on at least five different imaging positions along the RL interface. Elastic modulus calibration was performed using a reference value of 40 GPa for untreated copper (CF and FF). Two main assumptions were made: (1) the AFM tip could be modelled as a flat punch with radius R [13], and (2) the elastic modulus of copper remains effectively unchanged after thermal treatment. To validate the reliability of the modulus profiles, corresponding topography profiles were simultaneously analyzed to ensure that the RL region was flat and free from topographical artifacts (e.g., peaks or valleys), which could bias the modulus estimation.

**Figure 3:**
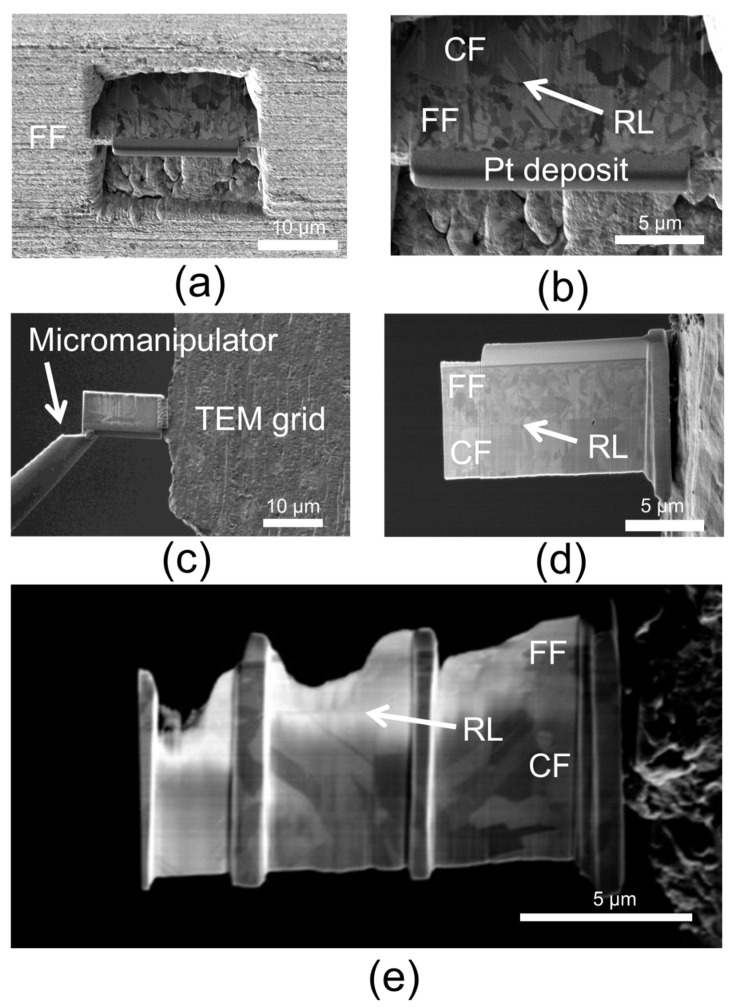
FIB-SEM lamella preparation procedure for TEM analysis of the DTH-Cr cross-section: (**a**) Trench milling on a DTH-Cr sample, pre-polished on the FF side; (**b**) Zoomed-in view after the first thinning step (to ~1 µm), showing the platinum (Pt) protective layers and the distinct grain morphologies of copper in the FF and CF; (**c**) Undercutting and lift-out of the lamella using a micromanipulator, followed by welding onto a TEM grid; (**d**) Zoom-in view of the ~1 µm-thick lamella, highlighting the FF, RL, and CF layers; (**e**) Final thinning of the lamella to a thickness of 50–100 nm.

Transmission electron microscopy (TEM) analysis was carried out on cross-sections of the DTH-Cr sample using a JEOL JEM-F200 TEM (JEOL, Tokyo, Japan) equipped with a cold field emission gun (cold FEG), operating at an accelerating voltage of 200 kV. Cross-sectional samples were prepared using the lift-out technique with a Helios Nanolab 650 focused ion beam–scanning electron microscope (FIB-SEM) system from FEI (Waltham, MA, USA). The sequential FIB-SEM preparation steps are illustrated in Figure 3. To reduce FIB-SEM preparation time, the functional foil (FF) side of the sample was first mechanically polished. To protect the surface from ion beam-induced damage, two platinum (Pt) protective layers were deposited on the FF: a ~200 nm layer using an electron beam at 3 kV and a ~1.5 µm layer using an ion beam at 30 kV (Figure 3a,b). Trenches were then milled around a thin lamella using the 30 kV ion beam, followed by an initial thinning to approximately 1 µm (Figure 3a). The lamella was undercut and lifted out using a micromanipulator (Figure 3c), then welded onto a copper TEM grid. It was further thinned to ~300 nm (Figure 3c,d). Final thinning was carried out to reach a thickness between 50 nm and 100 nm using a 30 kV ion beam, followed by low-energy thinning (5 kV) and final cleaning steps at 2 kV and 1 kV (Figure 3e). Conventional TEM imaging was used to observe the overall morphology of the sample. The crystalline nature of the RL was investigated through high-resolution TEM (HRTEM) imaging combined with fast Fourier transform (FFT) analysis. Elemental mapping in the region surrounding the RL was performed using energy-dispersive X-ray spectroscopy (EDS) in scanning TEM (STEM) mode.

## 3. Results and Discussion

### 3.1. Peeling Testing

The effect of thermal exposure on the release strength of DTH-Cr is summarized in Table 1. The release strength increases significantly from 5.9 N/m for the reference sample to 163 N/m after 110 min at 230 °C. This result indicates that the RL no longer functions as a peelable layer after thermal exposure, as a typical upper limit for peelability is 100 N/m [1]. A sharp increase in release strength is observed as the thermal exposure temperature rises from 170 °C to 230 °C, as shown in Table S1 of the Supplementary Data. To account for this increase in release strength with thermal exposure, the following hypotheses are proposed.

First, the RL may have partially crystallized, leading to increased atomic interactions with the crystalline Cu in the foils and, consequently, stronger bonding between the RL and the two Cu foils. It has been shown that amorphous Cr in electroplated layers begins to crystallize into metallic Cr at temperatures between 200 °C and 300 °C, a process associated with increased hardness of the Cr layer. Notably, no additional crystalline phases are expected to form within this temperature range [14,15]. This increase in hardness may also manifest as an increase in the RL’s elastic modulus, thereby reducing the initial modulus gradient between the crystalline Cu foils and the amorphous RL. A smaller elastic modulus mismatch could minimize stress localization at the RL during peeling, leading to a higher release strength. These hypotheses can be tested through TEM nanodiffraction and AFM analysis in nanomechanical mode.

Second, highly localized Cr–Cu interdiffusion may occur at the RL–Cu interfaces during thermal exposure at low temperatures. Cr atoms could diffuse into the Cu foil via copper grain boundaries, while Cu atoms may diffuse into the amorphous Cr layer. However, in both cases, diffusion is likely confined to the interfacial regions, with negligible activity in the bulk. This is consistent with the known behavior of the Cr–Cu system, where no intermetallic compound formation occurs below 750 °C, and the mutual bulk solubilities are very low (<0.2 at% for Cr in Cu and <0.1 at% for Cu in Cr) [16]. Among these processes, Cr diffusion into Cu is expected to be more significant than Cu diffusion into Cr at the interface [17]. Such localized interdiffusion could enhance bonding between the RL and the Cu foils, thereby increasing the release strength. However, confirming this hypothesis is particularly challenging, as it requires atomic-resolution analytical techniques, which were not available during this study. Consequently, this hypothesis remains unverified in the present work.

Lastly, exposing copper to air at temperatures above 150 °C can overcome the diffusion barrier posed by its native oxide passivation layer, which is only a few nanometers thick [18]. This results in the growth of the oxide layer and facilitates the diffusion of O_2_ from the air into the Cu. According to [19,20,21], oxygen diffusion primarily occurs via grain boundaries, with vacancy- and dislocation-assisted diffusion serving as important secondary pathways. Note that bulk diffusion is also possible but very limited. In the context of ultrathin Cu foils, this oxygen diffusion may reach the RL and react with the amorphous Cr, leading to the formation of a Cr oxide layer in the interfacial region between the FF and the RL. Understanding the influence of a Cr oxide layer on the adhesion between the two copper foils is not straightforward. Molecular dynamics (MD) simulations have shown that the presence of an oxide layer at the interface between a metal and an amorphous material enhances adhesion. This effect is attributed to stronger chemical bonding between the oxides and the amorphous material compared to that between the metal and the amorphous material [22]. However, another MD study reports the opposite result, indicating that interfacial oxides reduce the adhesion between a metal and an amorphous material [23]. Additionally, a separate study observes reduced interfacial strength but increased ductility in the presence of interfacial oxides—although no quantitative adhesion values were provided. The enhanced ductility is attributed to increased interfacial roughness caused by the oxides, which impeded the propagation of shear transformation zones [24]. It is also important to note that most likely, the formation of Cr oxides is not accompanied by the formation of voids; otherwise, the release strength would have dropped as noted in a previous work [25]. To gain a deeper understanding of the influence of interfacial oxides on the adhesion between metallic Cu and the amorphous Cr layer, a dedicated and comprehensive study is needed. Molecular dynamics (MD) simulations could serve as a valuable tool for this purpose. XPS measurements on the debonded surfaces of the DTH-Cr, along with STEM-EDS elemental analysis on the DTH-Cr cross-section, were conducted to verify the presence and distribution of oxygen species. While a native passivation layer may exist on the air-exposed surfaces of the DTH-Cr, no Cu oxide passivation layers are expected at the interfaces between the carrier foil (CF) and the release layer (RL), or between the functional foil (FF) and the RL. This is because the RL is typically deposited on the CF immediately following an acid cleaning treatment, and the Cu FF is directly electrodeposited onto the RL surface [3].

### 3.2. XPS Analysis

The elemental composition of the debonded surfaces of DTH-Cr (Figure 1b) is presented in Table 2, Table 3 and Table 4. A key observation is that, regardless of thermal exposure, the DTH-Cr-CF samples contain significantly more Cr and much less Cu than the DTH-Cr-FF samples. Only a small amount of the release layer (RL) remains adhered to the FF after peeling, indicating that debonding primarily occurs near the interface between the FF and RL. Since most of the Cr is found on the CF side, it is assumed that the most representative data on the RL composition comes from the DTH-Cr-CF samples. Consequently, the FF side (DTH-Cr-FF) is not further analyzed in this study for structural information about the RL. In the untreated DTH-Cr-CF samples, the dominant elements are C, Cr, and O, with Cu, N, and P present in smaller amounts (0.7 to 3.3 at%). The detected carbon is likely due to contamination from air exposure after peeling. The presence of Cu is probably related to regions where the RL is only a few nanometers thick, allowing detection of the underlying CF. Phosphorus may originate from the electrolyte bath used during RL electroplating. Elements with concentrations below 0.5 at% are excluded from analysis, as this represents the detection limit of the instrument used. To better analyze the evolution of oxygen in the release layer (RL), its content was normalized to the combined content of Cr and Cu, as shown in Table 2. This approach is based on the assumption that the total Cr and Cu concentrations in the RL remain approximately constant during thermal exposure, despite potential local interdiffusion at the interface. The resulting O/(Cr + Cu) ratio increases from 1.79 in the untreated DTH-Cr-CF sample to 2.48 after thermal exposure at 230 °C, indicating a significant rise in oxygen content within the RL and suggesting an oxidation process. To investigate whether this oxidation is correlated with exposure temperature, XPS measurements were conducted at intermediate temperatures of 170 °C and 200 °C (see Supplementary Table S2). The O/(Cr + Cu) ratio increases progressively with temperature in the following order: untreated DTH-Cr-CF (1.79) < thermally exposed DTH-Cr-CF at 170 °C (2.11) < thermally exposed DTH-Cr-CF at 200 °C (2.42) < thermally exposed DTH-Cr-CF at 230 °C (2.48). This trend confirms that oxidation in the RL intensifies with increasing thermal exposure.

**Figure 4:**
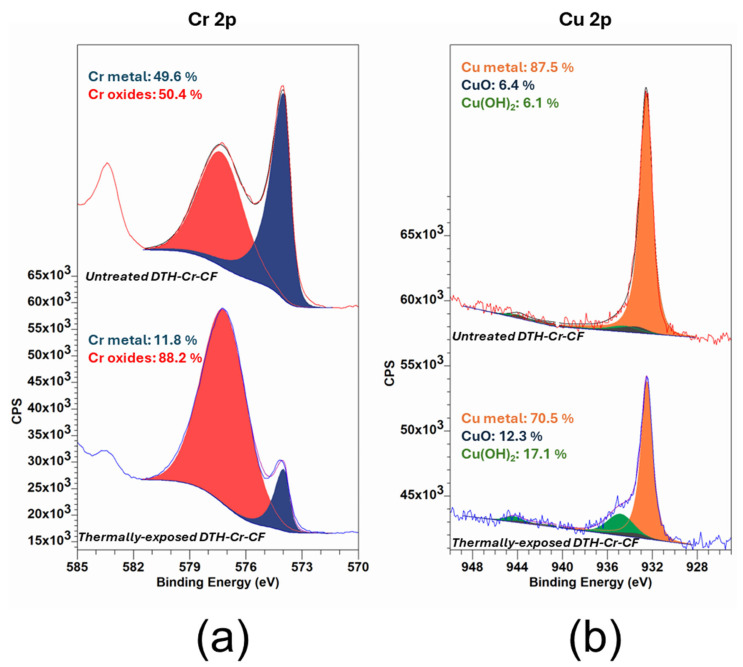
XPS peak deconvolution of Cr 2p (**a**) and Cu 2p (**b**) in the case of untreated DTH-Cr-CF and thermally exposed DTH-Cr-CF at 230 °C.

Two main forms of chromium have been identified in the DTH-Cr-CF samples based on the analysis of Cr 2p peaks (Table 3 and Figure 4a): metallic chromium (Cr^0^) and chromium oxides. According to the literature, amorphous electroplated Cr typically contains CrO_3_ and Cr_2_O_3_. CrO_3_ is stable below 250 °C, while Cr_2_O_3_ remains stable up to 2400 °C, indicating that no decomposition of these potential oxides is expected under the thermal exposure conditions used in this study [26]. It is also well established that a passivation layer forms spontaneously and rapidly on the surface of amorphous Cr upon exposure to air [27,28]. This is applicable to both the DTH-Cr-CF and DTH-Cr-FF surfaces after the peeling process. As a result, the detected Cr oxides may include both the native passivation layer and oxides formed within the bulk of the RL during thermal exposure. Thermal treatment induces significant oxidation of metallic Cr: the Cr^0^ content decreases markedly from 49.6% in the untreated state to 11.8% after exposure, accompanied by a corresponding increase in chromium oxide content. To validate this trend, additional thermal exposure conditions were examined (Table S3). A clear correlation was observed between increasing exposure temperature and the progressive transformation of Cr^0^ into Cr oxides. This oxidation process is consistent with the diffusion of atmospheric O_2_ into the RL, likely facilitated through Cu grain boundaries. Further insight into the underlying mechanism is expected from ongoing TEM analyses.

Since the interface between DTH-Cr-CF and the release layer (RL) was not exposed to the atmosphere after thermal treatment—even following the peeling process—the chemical states of copper on the CF side were analyzed by examining the Cu 2p peak (Table 4 and Figure 4b). Before thermal exposure, the identified copper species are metallic copper (Cu^0^) at 87.5%, copper(II) hydroxide (Cu(OH)_2_) at 6.1%, and copper(II) oxide (CuO) at 6.4%. The presence of CuO suggests that not all copper was reduced to its metallic state during the electrodeposition of the CF. Meanwhile, the detection of Cu(OH)_2_ is attributed to the partial reaction of copper oxides with water from the electrolytic bath or with atmospheric moisture [29]. After thermal exposure at 230 °C, a decrease in metallic copper (Cu^0^) content from 87.5% to 70.5% is observed, accompanied by an increase in Cu(OH)_2_ from 6.1% to 17.1% and in CuO from 6.4% to 12.3%. However, these trends have not been consistently confirmed at other thermal exposure temperatures, as shown in Table S4. This variability may stem from the aleatory nature of the peeling region within the RL, which can shift with thermal treatment. Consequently, the amount of copper detected by XPS beneath the RL can also vary unpredictably, making it difficult to draw definitive conclusions. Nevertheless, it is noteworthy that the increase in release strength of DTH-Cr after thermal exposure may be linked to enhanced interactions between the growing concentration of chromium oxides in the RL and CuO present in the copper foil. These evolving interfacial chemistries could play a significant role in the adhesion or mechanical properties observed post-treatment.

### 3.3. AFM Analysis

The evolution of the elastic modulus and thickness of the RL for both untreated DTH-Cr and DTH-Cr thermally exposed at 230 °C is presented in Table 5. Minimal or no changes are observed in these structural parameters upon thermal exposure, a trend that is consistent with results at other exposure temperatures (Table S5). This suggests that no significant formation of rigid crystalline phases—such as crystalline Cr or Cr oxides—occurs within the RL that would otherwise alter the elastic modulus of the initially amorphous structure. Therefore, the Cr oxides detected by XPS in the RL are presumed to remain in an amorphous state. Regarding thickness, an increase was anticipated if a passivation oxide layer had formed at the CF–RL or FF–RL interfaces, as suggested in previous studies [30]. However, no such increase was detected by AFM, indicating that any oxide layer formed is either too thin to be resolved or not present in measurable quantities. The evaluation of both the elastic modulus and RL thickness using AFM nanomechanical testing is subject to two key limitations that affect spatial resolution. First, despite the very shallow indentation depths achieved in AM-FM mode, the measured modulus at a given point is influenced by adjacent phases due to the presence of a mechanically affected zone [31]. In this context, the RL thickness—determined by TEM to be approximately 5–10 nm (see next section)—must be compared to the maximum indentation depth of roughly 1 nm. Second, assuming the AFM tip behaves as a flat punch, its effective contact diameter is approximately 14 nm (based on a nominal tip diameter of 7 nm for AC160 probes). This implies that when probing the RL, the tip is likely in simultaneous contact with adjacent copper phases (either CF or FF). Together, these effects limit the sensitivity of AFM measurements and make it challenging to detect subtle changes in modulus or thickness of the RL with high confidence.

### 3.4. TEM Analysis

**Figure 5:**
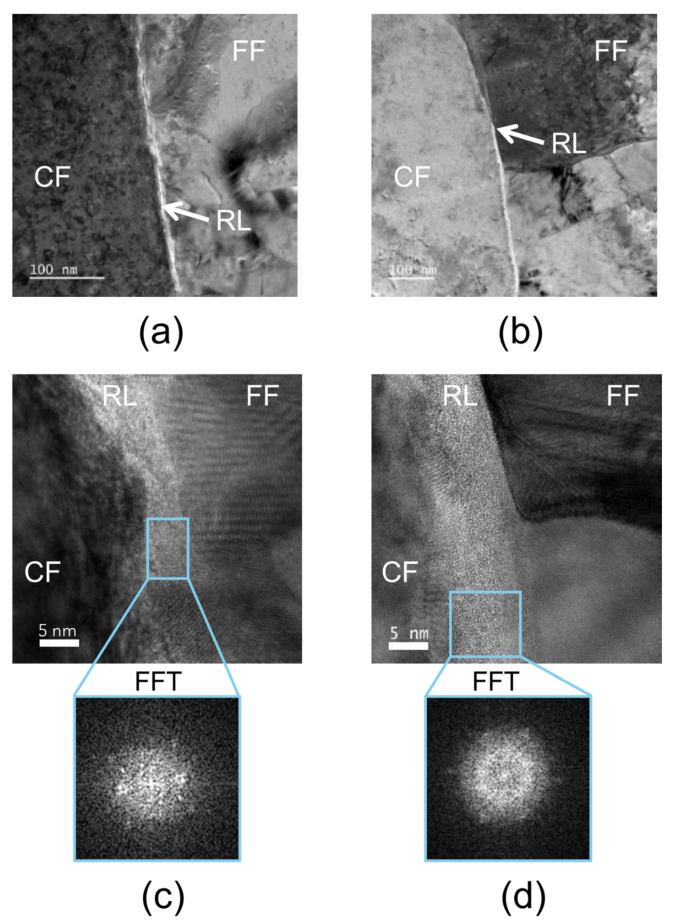
TEM micrographs (**a**,**b**) and HRTEM micrographs with fast Fourier transform (FFT) of an RL area (**c**,**d**), in the case of untreated DTH-Cr (**a**,**c**) and thermally exposed DTH-Cr at 230 °C (**b**,**d**).

**Figure 6:**
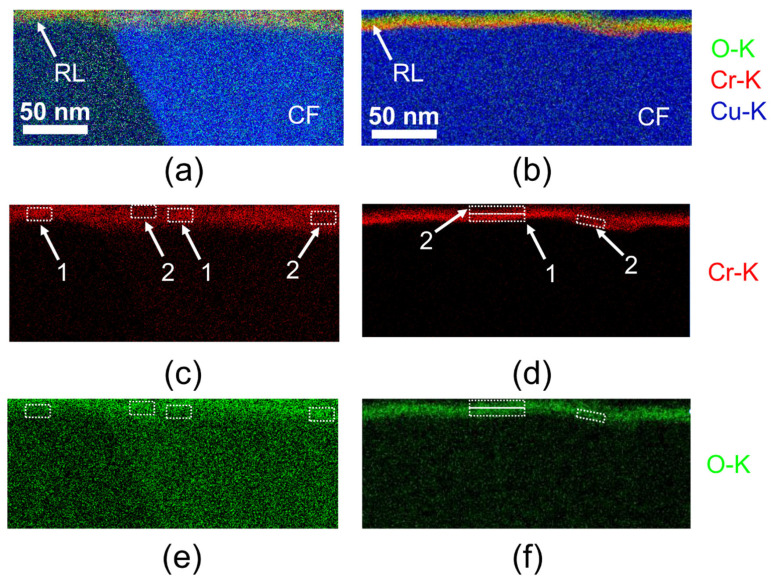
EDS mapping in the case of untreated DTH-Cr (**a**,**c**,**e**) and thermally exposed DTH-Cr at 230 °C (**b**,**d**,**f**), focused on the detection of O-K, Cr-K, and Cu-K (1: area with a high Cr concentration, and 2: area with a low Cr concentration).

TEM investigations in HRTEM mode were conducted on cross-sections of both untreated and thermally exposed DTH-Cr samples, as shown in Figure 5. At low magnification (Figure 5a,b), the RL is visible as a region of bright contrast situated between the two copper foils. At higher magnification (Figure 5c,d), clear morphological differences emerge between the untreated and thermally exposed samples: the RL appears flatter and more homogeneous following thermal treatment at 230 °C. The reasons for these observations remain uncertain. However, one hypothesis suggests that prior to thermal exposure, FIB milling may interact with the release of internal stresses in the RL, leading to local distortions observed during TEM imaging [32]. Following thermal exposure, these internal stresses may be further relieved, resulting in a smoother surface after FIB preparation. In both conditions, fast Fourier transform (FFT) analyses of selected RL regions reveal two main features: (i) a diffuse intensity pattern, confirming the amorphous nature of the RL, and (ii) residual bright diffraction spots, which are attributed to the crystalline copper from both the carrier foil (CF) and the functionalized foil (FF) adjacent to the RL (Figure 5c,d). The absence of crystalline phases in the RL after thermal exposure, as indicated by FFT analysis of HRTEM micrographs, is consistent with the AFM measurements of elastic modulus (Table 5), which show no significant change suggestive of crystallization. Elemental mapping of untreated and thermally exposed DTH-Cr samples is presented in Figure 6. The combined Cr and O maps (Figure 6a) show a quite homogeneous distribution of both elements throughout the RL. It is important to note that oxygen may be present both as dissolved O_2_ and in the form of oxides. However, when examining the individual Cr maps (Figure 6c,e), localized regions of lower Cr concentration are observed. These regions correspond to areas of higher oxygen concentration, suggesting local Cr oxidation within the RL. The regions with low Cr concentration are interpreted as Cr oxide-rich areas, while the regions with higher Cr concentration correspond to metallic Cr^0^, as supported by the data in Table 3. After thermal exposure, the spatial distributions of Cr and O show noticeable changes (Figure 6b). Specifically, a distinct layer with reduced Cr concentration forms in the interfacial region between the FF and the RL, while the remainder of the RL exhibits higher Cr concentrations (Figure 6d). In contrast, the oxygen distribution remains relatively uniform throughout both the low-Cr interfacial layer and the rest of the RL (Figure 6f). Based on XPS results, thermal exposure leads to the transformation of metallic chromium (Cr^0^) into chromium oxides, which become the dominant species (Table 3). This oxidation process is likely most active at the FF–RL interface, facilitated by the diffusion of O_2_ from the air through copper grain boundaries. Since the copper grain size in the functionalized foil (FF) is smaller than that in the carrier foil (CF), oxygen diffusion is expected to be more pronounced from the FF toward the RL than from the CF side (Figure 2b,d). Accordingly, the low-Cr concentration layer observed at the FF–RL interface is interpreted as a chromium oxide layer. In the rest of the RL, the Cr composition is expected to remain close to its initial state prior to heat treatment (Table 3). Following thermal exposure, the oxygen concentration within the RL has likely become more homogeneous, existing both as dissolved O_2_ and as oxides. This homogenization makes it difficult to distinguish between Cr oxide and Cr^0^ regions, unlike the clearer differentiation observed prior to thermal treatment (Figure 6e).

### 3.5. Summary of the Mechanisms

The combined analysis of debonded DTH-Cr-FF and DTH-Cr-CF foils by XPS, together with cross-sectional AFM and TEM investigations, reveals the structural transformations of DTH-Cr after thermal exposure at 230 °C in air. The primary change is the oxidation of the release layer (RL), resulting in the formation of a chromium oxide-rich layer in the interfacial region between the functionalized foil (FF) and the RL. Above 150 °C, the native passivation oxide layer on the copper foil surfaces no longer acts as an effective diffusion barrier, allowing O_2_ from the air to penetrate into the DTH-Cr structure. Oxygen may diffuse preferentially through the grain boundaries of the copper FF, which has small grains, until reaching the RL. In contrast, the large grain size of the copper carrier foil (CF) may limit O_2_ diffusion from that side. Note that the potential thickening of the Cu passivation layer on foil surfaces was not investigated in this study. Upon reaching the amorphous Cr in the RL, oxygen oxidizes Cr, forming an amorphous chromium oxide layer localized in the FF–RL interfacial region. This is supported by XPS data showing an increase in Cr oxide species and a decrease in metallic Cr^0^, as well as STEM-EDS elemental mapping. An Ellingham diagram was constructed showing the Gibbs free energy changes associated with the oxidation of Cr and Cu in the presence of oxygen (see Figure S1 in the Supplementary Data) [33]. The evolution of Gibbs free energy was plotted as a function of temperature for the various possible oxidation reactions of Cr and Cu. At 230 °C, the most thermodynamically favorable reaction for Cr is its oxidation to Cr_2_O_3_ (−659.5 kJ/mol), followed by oxidation to Cr_3_O_4_ (−611.6 kJ/mol), and then to CrO (−602.6 kJ/mol). Overall, the oxidation of Cr is more favorable than that of Cu. For Cu, the lowest Gibbs free energy corresponds to the formation of Cu_2_O (−266.6 kJ/mol), followed by CuO (−219.5 kJ/mol) and CuO_2_ (−196.8 kJ/mol). AFM and electron diffraction confirm that the RL remains amorphous. One intriguing finding is that HRTEM potentially reveals the release of internal stress within the RL after thermal exposure, as evidenced by the surface of the RL becoming flatter and more homogeneous. While local interdiffusion of Cr atoms into the Cu foils and Cu atoms into the amorphous RL via grain boundaries cannot be ruled out, it was not demonstrated in this work.

**Figure 7:**
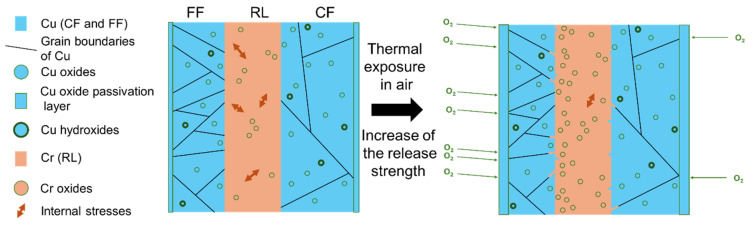
Schematic representation of the influence of thermal exposure (230 °C) in air on the structural properties of DTH-Cr (note that the thicknesses of the FF, RL, and CF are not representative of the reality).

The formation of a Cr oxide layer likely enhances the interaction between the RL and the FF, as oxides are present on both interfaces. Additionally, any Cr–Cu atomic interdiffusion may further strengthen the adhesion between the RL and the copper foils. The release of internal stresses following thermal exposure may also contribute to a reduced RL peelability. Together, these three mechanisms provide a plausible explanation for the observed increase in release strength after thermal exposure. A summary of these findings and hypotheses is illustrated in Figure 7. It is important to note that nanoscale measurements (TEM and AFM) were conducted across several cross-sectional areas to assess the consistency of these localized tests. However, the collected data may not be representative of the entire RL, which spans meter-scale lateral dimensions.

The proposed methodology may be applied to any nanometric adhesive or release layer [12] and, more broadly, to any nanometric interlayer involved in material assembly [34,35], offering a wide range of potential applications across electronics and semiconductors, energy and photovoltaics, and optics and photonics.

## 4. Conclusions

In this paper, we developed a methodology to analyze the structural properties of a nanometer-thick release layer situated between two micrometer-thick copper foils—an industrial material commonly used for conductive tracks in electronics. The approach integrates peel-off testing of the full assembly, XPS analysis of debonded surfaces, and AFM and TEM investigations of the cross-section. This combined methodology was applied before and after thermal exposure at 230 °C in air to characterize the structural evolution induced by the heat treatment.

Thermal exposure leads to a significant increase in the release strength of DTH-Cr, rising from 5.9 N/m before treatment to 163 N/m afterward.

XPS analysis of the release layer (RL) reveals that the content of amorphous metallic chromium (Cr^0^) decreases substantially from 49.6% to 11.8% (−76.2%), while the proportion of amorphous chromium oxides increases from 50.4% to 88.2% (+42.9%). This transformation is attributed to oxidation of Cr^0^ driven by O_2_ diffusion from the air into the RL via copper grain boundaries.

TEM observations confirm the presence of a Cr oxide layer localized at the interface between the functionalized foil (FF) and the RL, which is absent at the carrier foil (CF)–RL interface. The smaller grain size in the FF compared to the CF likely facilitates oxygen diffusion through the FF’s grain boundaries, whereas O_2_ diffusion from the CF side is minimal.

Electron diffraction and AFM measurements indicate no crystallization occurs within the RL after thermal exposure, confirming that the Cr oxide layer remains amorphous.

HRTEM imaging indicates that after thermal exposure, the RL becomes flatter and more homogeneous. The literature shows that FIB surface preparation can activate the relief of internal stresses, which appear as material distortions under microscopy. Accordingly, thermal exposure is believed to relieve these internal stresses.

The enhanced release strength is attributed to stronger bonding at the FF–RL interface, resulting from interactions between Cu oxides in the FF and the Cr oxide layer in the RL. Additionally, localized atomic interdiffusion of Cr and Cu atoms at the RL–Cu interfaces—Cr diffusing along Cu grain boundaries and Cu atoms penetrating the amorphous RL network—may further contribute. Confirming this interdiffusion will require chemical analysis techniques with atomic spatial resolution, such as, for example, atomic probe tomography, which could be addressed in future work. Last, the elimination of internal stress can reduce the peelability of the RL, increasing the release strength. To gain deeper insight into the structural transformations of the RL during thermal exposure and their effect on the release strength, a comprehensive and systematic investigation using molecular dynamics simulations would be highly valuable.

This study provides new insights into the thermal stability of DTH products, which can support the development of improved designs—such as incorporating enhanced oxygen diffusion barriers to limit oxidation of the release layer.

## Figures and Tables

**Table 1:** Average release strength of the untreated and thermally exposed DTH-Cr measured by 90° peeling testing.

Sample	Release Strength (N/m)
Untreated DTH-Cr	5.9
Thermally exposed DTH-Cr at 230 °C	163.0

**Table 2:** Elemental composition of the untreated and thermally exposed DTH-Cr as determined by XPS.

Sample	Elemental Composition									
	C (at%)	Cr (at%)	Cu (at%)	N (at%)	Na (at%)	O (at%)	P (at%)	S (at%)	Si (at%)	O/(Cr + Cu) (-)
Untreated DTH-Cr-CF	27.8	22.2	2.0	0.7	<0.5	43.3	3.3	<0.5	<0.5	1.79
Untreated DTH-Cr-FF	31.7	1.8	33.9	<0.5	<0.5	30.1	2.4	<0.5	<0.5	0.84
Thermally exposed DTH-Cr-CF at 230 °C	21.1	20.0	1.2	<0.5	0.6	52.5	3.5	<0.5	<0.5	2.48

**Table 3:** Cr species composition of the untreated and thermally exposed DTH-Cr-CF as determined by XPS.

Sample	Composition in Cr Species from Cr 2p	
	Cr^0^ (%)	Cr Oxides (%)
Untreated DTH-Cr-CF	49.6	50.4
Thermally exposed DTH-Cr-CF at 230 °C	11.8	88.2

**Table 4:** Cu species composition of the untreated and thermally exposed DTH-Cr-CF as determined by XPS.

Sample	Composition in Cu Species from Cu 2p		
	Cu^0^ (%)	Cu(OH)_2_ (%)	CuO (%)
Untreated DTH-Cr-CF	87.5	6.1	6.4
Thermally exposed DTH-Cr-CF at 230 °C	70.5	17.1	12.3

**Table 5:** Elastic modulus and thickness of the RL in the case of the untreated and thermally exposed DTH-Cr-CF as determined by AFM.

Sample	AFM Analysis of RL	
	Minimum Elastic Modulus (GPa)	Thickness (nm)
Untreated DTH-Cr	27.1 ± 8.2	13.8 ± 2.8
Thermally exposed DTH-Cr at 230 °C	27.2 ± 5.1	15.3 ± 8.5

## Data Availability

The original contributions presented in the study are included in the article, further inquiries can be directed to the corresponding author.

## Supplementary Materials

The following supporting information can be downloaded at: https://www.mdpi.com/article/10.3390/ma18143316/s1, Table S1: Average release strength of the untreated and thermally-exposed DTH-Cr measured by 90° peeling testing. Table S2: Elemental composition of the untreated and thermally-exposed DTH-Cr at 170 °C, 200 °C, and 230 °C for 110 min, as determined by XPS. Table S3: Cr species composition of the untreated and thermally-exposed DTH-Cr-CF at 170 °C, 200 °C, and 230 °C for 110 min, as determined by XPS. Table S4: Cu species composition of the untreated and thermally-exposed DTH-Cr-CF at 170 °C, 200 °C, and 230 °C for 110 min, as determined by XPS. Table S5: Elastic modulus and thickness of the RL of the untreated and thermally-exposed DTH-Cr at 170 °C, 200 °C, and 230 °C for 110 min, as determined by AFM. Figure S1: Ellingham diagram of Cu and Cr generated from the Dissemination of IT for the Promotion of Materials Science (DoITPoMS) website, Cambridge University (UK) [36].
