# Supplementary material for: Method for Elucidating the Structural Evolution of a Nanoscale Release Layer in Double Copper Foils Under Thermal Exposure
Source: Materials (Basel). 2025 Jul 14;18(14):3316. doi: 10.3390/ma18143316 (PMC12298920; doi:10.3390/ma18143316)
Supplement: Supplementary file 1 [file materials-18-03316-s001.zip › materials-3737577-supplementary.pdf]

Supplementary data

Method for Elucidating the Structural Evolution of a Nanoscale Release Layer in Double Copper Foils under Thermal Exposure

Rutuja Bhusari [1], Julien Bardon [1], Jérôme Guillot [2], Adrian-Marie Philippe [2], Sascha Scholzen [3], Zainhia Kaidi [3] and Frédéric Addiego [1]

[1]Structural Composites Unit, Luxembourg Institute of Science and Technology, 5 Avenue des Hauts-Fourneaux, L-4362 Esch-sur-Alzette, Luxembourg
[2]Advanced Analyses and Support Unit, Luxembourg Institute of Science and Technology, 5 Avenue des Hauts-Fourneaux, L-4362 Esch-sur-Alzette, Luxembourg
[3]Circuit Foil Luxembourg, 6 Salzbaach, L-9559 Wiltz, Luxembourg

Table S1. Average release strength of the untreated and thermally-exposed DTH-Cr measured by 90° peeling testing.

Sample	Release strength (N/m)
Untreated DTH-Cr	5.9
Thermally-exposed DTH-Cr at 170°C	26.0
Thermally-exposed DTH-Cr at 200°C	53.8
Thermally-exposed DTH-Cr at 230°C	163.0

Table S2. Elemental composition of the untreated and thermally-exposed DTH-Cr at 170°C, 200°C, and 230°C for 110 min, as determined by XPS.

Sample	Elemental composition									
	C (at%)	Cr (at%)	Cu (at%)	N (at%)	Na (at%)	O (at%)	P (at%)	S (at%)	Si (at%)	O (Cr + Cu) (-)
Untreated DTH-Cr-CF	27.8	22.2	2.0	0.7	<0.5	43.3	3.3	0.7	<0.5	1.79
Thermally-exposed DTH-Cr-CF at 170°C	26.1	21.4	1.0	<0.5	<0.5	47.3	2.5	0.6	0.6	2.11
Thermally-exposed DTH-Cr-CF at 200°C	24.1	19.6	1.2	<0.5	<0.5	50.3	2.9	<0.5	1.0	2.42
Thermally-exposed DTH-Cr-CF at 230°C	21.1	20.0	1.2	<0.5	0.6	52.5	3.5	<0.5	<0.5	2.48

Table S3. Cr species composition of the untreated and thermally-exposed DTH-Cr-CF at 170°C, 200°C, and 230°C for 110 min, as determined by XPS.

Sample	Composition in Cr species from Cr 2p	
	Cr^0 (%)	Cr oxides (%)
Untreated DTH-Cr-CF	49.6	50.4
Thermally-exposed DTH-Cr-CF at 170°C	32.8	67.2
Thermally-exposed DTH-Cr-CF at 200°C	22.9	77.1
Thermally-exposed DTH-Cr-CF at 230°C	11.8	88.2

Table S4. Cu species composition of the untreated and thermally-exposed DTH-Cr-CF at 170°C, 200°C, and 230°C for 110 min, as determined by XPS.

Sample	Composition in Cu species from Cu 2p		
	Cu^0 (%)	$Cu(OH)_2$ (%)	CuO (%)
Untreated DTH-Cr-CF	87.5	6.1	6.4
Thermally-exposed DTH-Cr-CF at 170°C	89.5	10.1	0.5
Thermally-exposed DTH-Cr-CF at 200°C	89.0	11.0	0.0
Thermally-exposed DTH-Cr-CF at 230°C	70.5	17.1	12.3

Table S5. Elastic modulus and thickness of the RL of the untreated and thermally-exposed DTH-Cr at 170°C, 200°C, and 230°C for 110 min, as determined by AFM.

Sample	AFM analysis of RL	
	Minimum elastic modulus (GPa)	Thickness (nm)
Untreated DTH-Cr	27.1 ± 8.2	13.8 ± 2.8
Thermally-exposed DTH-Cr at 170°C	22.7 ± 4.4	10.6 ± 2.6
Thermally-exposed DTH-Cr at 200°C	19.7 ± 6.0	13.2 ± 6.5
Thermally-exposed DTH-Cr at 230°C	27.2 ± 5.1	15.3 ± 8.5

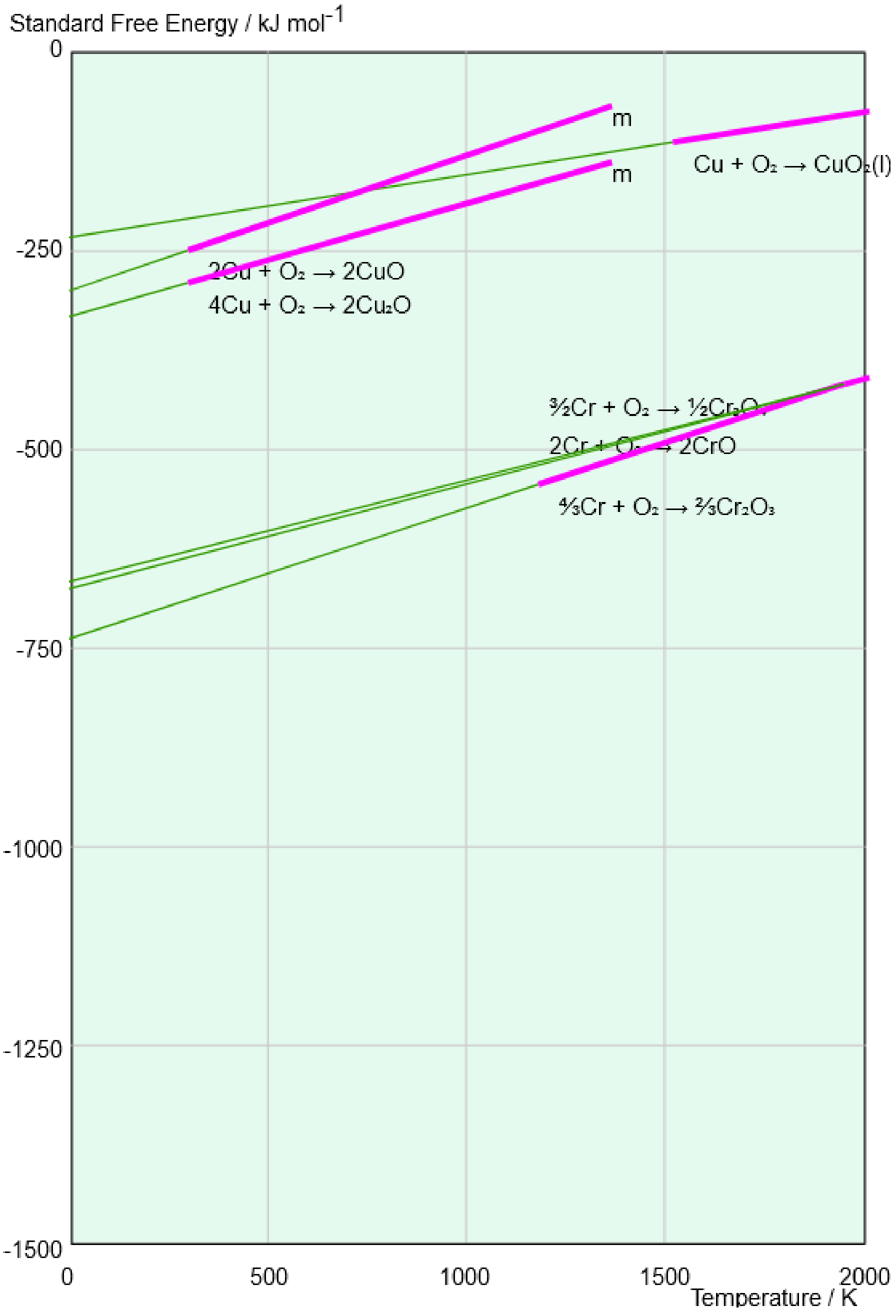

Figure S1. Ellingham diagram of Cu and Cr generated from the Dissemination of IT for the Promotion of Materials Science (DoITPoMS) website, Cambridge University (UK) [1].

Reference:

[1] Available online: https://www.doitpoms.ac.uk/tlplib/ellingham_diagrams/interactive.php (accessed on 11 June 2025).
